# 4,5,8a-Triphenyl­perhydro­pyrimido[4,5-*d*]pyrimidine-2,7-dione monohydrate

**DOI:** 10.1107/S1600536810019525

**Published:** 2010-05-29

**Authors:** Yulin Zhu, Wanhong Qiu, Fufen Yang, Guichan Li

**Affiliations:** a School of Chemistry and Environment, South China Normal University, Guangzhou 510006, People’s Republic of China

## Abstract

The title compound, C_24_H_22_N_4_O_2_·H_2_O, was synthesized by the trimethyl­chloro­silane-catalysed reaction between urea, benzaldehyde and acetophenone. The organic mol­ecule comprises two fused tetra­hydro­pyrimidinone rings with phenyl substituents at the 4- and 5-positions on the tetra­hydro­pyrimidinone rings and a third phenyl substituent at the ring junction 8-position. The 4- and 5-substituted phenyl rings are inclined at a dihedral angle of 22.72 (11)° to one another and make angles of 47.95 (7) and 65.76 (7)° with the third phenyl substituent. In the crystal structure, inter­molecular N—H⋯O contacts link pyrimido[4,5-*d*]pyrimidine mol­ecules into centrosymmetric dimers. Additional N—H⋯O and O—H⋯O hydrogen bonds involving the water mol­ecule generate a three-dimensional network.

## Related literature

For the therapeutic and pharmacological properties of pyrimidopyrimidines, see: Agarwal *et al.* (2005 ▶); Gangjee *et al.* (2005 ▶). For the synthesis of related compounds, see: Shi *et al.* (2007 ▶); Zhu *et al.* (2005 ▶). For reference bond-length data, see Allen *et al.* (1987 ▶).
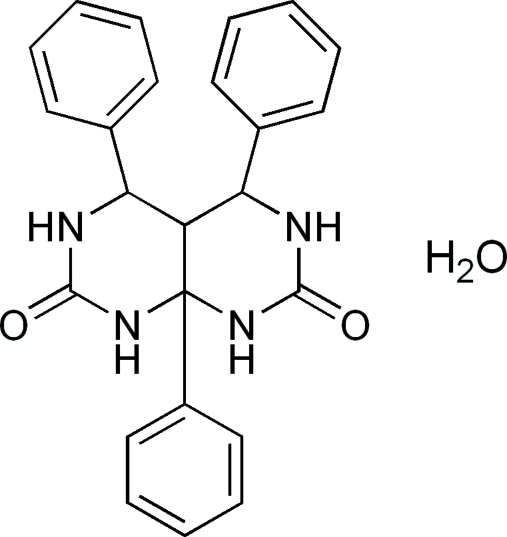


## Experimental

### 

#### Crystal data

C_24_H_22_N_4_O_2_·H_2_O
*M* *_r_* = 416.47
Monoclinic, 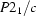

*a* = 11.3150 (2) Å
*b* = 17.4935 (3) Å
*c* = 10.5794 (2) Å
β = 94.731 (1)°
*V* = 2086.94 (6) Å^3^
*Z* = 4
Mo *K*α radiation
μ = 0.09 mm^−1^
*T* = 298 K
0.30 × 0.15 × 0.15 mm

#### Data collection

Bruker APEXII area-detector diffractometer
Absorption correction: multi-scan (*SADABS*; Bruker, 2004 ▶) *T*_min_ = 0.977, *T*_max_ = 0.981
18167 measured reflections
3768 independent reflections
2835 reflections with *I* > 2σ(*I*)
*R*_int_ = 0.035

#### Refinement

*R*[*F* ^2^ > 2σ(*F* ^2^)] = 0.040
*wR*(*F* ^2^) = 0.110
*S* = 1.04
3768 reflections
305 parameters
H atoms treated by a mixture of independent and constrained refinement
Δρ_max_ = 0.25 e Å^−3^
Δρ_min_ = −0.20 e Å^−3^

### 

Data collection: *APEX2* (Bruker, 2004 ▶); cell refinement: *SAINT* (Bruker, 2004 ▶); data reduction: *SAINT*; program(s) used to solve structure: *SHELXS97* (Sheldrick, 2008 ▶); program(s) used to refine structure: *SHELXL97* (Sheldrick, 2008 ▶); molecular graphics: *SHELXTL* (Sheldrick, 2008 ▶); software used to prepare material for publication: *SHELXTL*.

## Supplementary Material

Crystal structure: contains datablocks global, I. DOI: 10.1107/S1600536810019525/sj2783sup1.cif

Structure factors: contains datablocks I. DOI: 10.1107/S1600536810019525/sj2783Isup2.hkl

Additional supplementary materials:  crystallographic information; 3D view; checkCIF report

## Figures and Tables

**Table 1:** Hydrogen-bond geometry (Å, °)

*D*—H⋯*A*	*D*—H	H⋯*A*	*D*⋯*A*	*D*—H⋯*A*
N1—H5⋯O1^i^	0.87 (2)	2.05 (2)	2.9098 (19)	169.5 (18)
N2—H24⋯O2^ii^	0.862 (19)	2.16 (2)	2.9774 (19)	157.9 (16)
N3—H4⋯O3^ii^	0.93 (2)	1.87 (2)	2.787 (2)	168.1 (18)
O3—H1⋯O1^iii^	0.94 (3)	1.88 (3)	2.747 (2)	152 (3)
O3—H2⋯O2	0.87 (3)	1.99 (3)	2.751 (2)	146 (2)

Symmetry codes: (i) 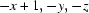; (ii) 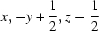; (iii) 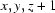.

**Fig. 1.**
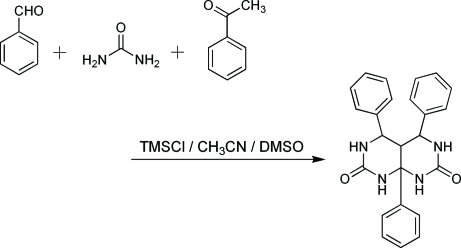
Trimethylchlorosilane (TMSCl) catalyzed synthesis of the title compound.

**Fig. 2.**
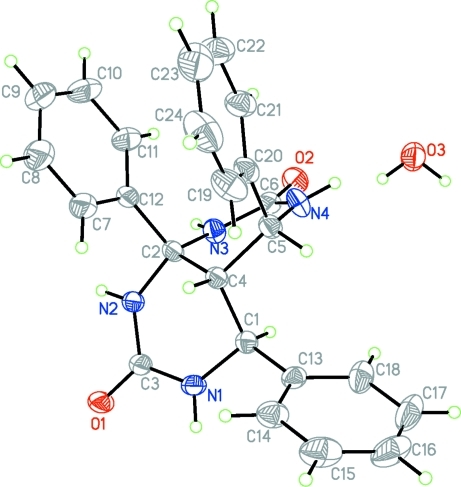
View of the title compound showing the atom–labelling scheme. Displacement ellipsoids are drawn at the 50% probability level. H atoms are presented as a small spheres of arbitrary radius.

**Fig. 3.**
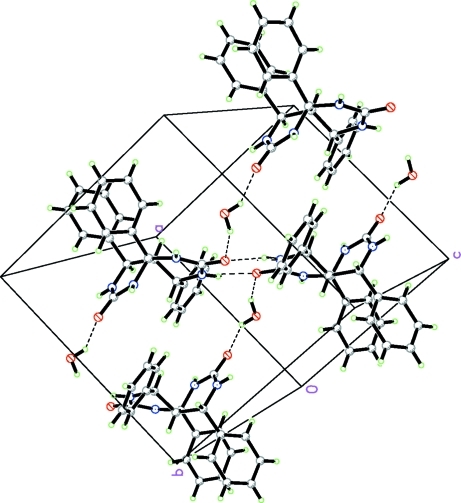
The crystal packing of the title compound (I).

## supplementary crystallographic information

### Comment

Pyrimidopyrimidine compounds have recently been paid much attention because of their therapeutic and pharmacological properties (Agarwal *et al.*, 2005; Gangjee *et al.*, 2005). As a part of our studies on the synthesis of the Biginelli-type compounds (Zhu *et al.*, 2005), the title compound was synthesized by a one-pot three-component reaction between acetophenone, urea and benzaldehyde in presence of trimethylchlorosilane as a catalyst in a yield of 86% (Fig. 1) (Shi *et al.*, 2007).

The bond lengths and angles in the molecule are normal (Allen *et al.*, 1987). The asymmetric unit contains a pyrimidopyrimidine molecule and a solvate water molecule (Fig. 2). The organic molecule comprises two fused tetrahydropyrimidinone rings with phenyl substituents at the 4, and 5 positions on the tetrahydropyrimidinone rings and a third phenyl substituent at the ring junction 8 position. The 4- and 5- substituted phenyl rings are inclined at a dihedral angle of 22.72 (0.11) to one another and make angles of 47.95(0.07) and 65.76(0.07) with the third phenyl substituent.The molecules in the structure are linked *via* intermolecular N1—H5···O1 and N2—H24···O2 hydrogen bonds. In addition, the molecule is connected to the water molecule by N3—H4···O3, O3—H1···O1 and O3—H2···O2 hydrogen bonds which generate a three dimensional network (Fig. 3).

### Experimental

Acetophenone (0.6 g, 5.0 mmol), urea (0.39 g, 6.5 mmol), benzaldehyde (0.53 g, 5.0 mmol), dimethyl sulfoxide (2.5 ml) and acetonitrile (5.0 ml) were mixed in a 25 ml flask and trimethylchlorosilane (0.54 g, 5.0 mmol) was added dropwise at room temperature (Fig. 1). Then the reaction mixture was stirred under 80°C for 5-6 h while a white precipitate was developing. The product was isolated by filtration through a Büchner funnel and washed first with water, then ethanol. The product was then dried to give a crystalline powder. Colourless, block-shaped single crystals of the title compound were obtained by slow evaporation from ethanol at room temperature.

### Refinement

The H atoms bound to C were positioned geometrically and allowed to ride on their parent atoms, with C—H = 0.93–0.98 Å and *U*_iso_ =1.2 or 1.5U_eq_(parent atom). H atoms bound to the N and water O atoms were found in a difference map and refined freely with isotropic displacement parameters.

### Crystal data

**Table:**

C_24_H_22_N_4_O_2_·H_2_O	*F*(000) = 880.0
*M**_r_* = 416.47	*D*_x_ = 1.326 Mg m^−^^3^
Monoclinic, *P*2_1_/*c*	Mo *K*α radiation, λ = 0.71073 Å
Hall symbol: -P 2ybc	Cell parameters from 5618 reflections
*a* = 11.3150 (2) Å	θ = 2.3–23.5°
*b* = 17.4935 (3) Å	µ = 0.09 mm^−^^1^
*c* = 10.5794 (2) Å	*T* = 298 K
β = 94.731 (1)°	Block, colourless
*V* = 2086.94 (6) Å^3^	0.30 × 0.15 × 0.15 mm
*Z* = 4	

### Data collection

**Table:**

Bruker APEXII area-detector diffractometer	3768 independent reflections
Radiation source: fine-focus sealed tube	2835 reflections with *I* > 2σ(*I*)
graphite	*R*_int_ = 0.035
φ and ω scans	θ_max_ = 25.2°, θ_min_ = 1.8°
Absorption correction: multi-scan (*SADABS*; Bruker, 2004)	*h* = −13→10
*T*_min_ = 0.977, *T*_max_ = 0.981	*k* = −20→20
18167 measured reflections	*l* = −12→12

### Refinement

**Table:**

Refinement on *F*^2^	Secondary atom site location: difference Fourier map
Least-squares matrix: full	Hydrogen site location: difference Fourier map
*R*[*F*^2^ > 2σ(*F*^2^)] = 0.040	H atoms treated by a mixture of independent and constrained refinement
*wR*(*F*^2^) = 0.110	*w* = 1/[σ^2^(*F*_o_^2^) + (0.0452*P*)^2^ + 0.5316*P*] where *P* = (*F*_o_^2^ + 2*F*_c_^2^)/3
*S* = 1.04	(Δ/σ)_max_ = 0.001
3768 reflections	Δρ_max_ = 0.25 e Å^−^^3^
305 parameters	Δρ_min_ = −0.20 e Å^−^^3^
0 restraints	Extinction correction: *SHELXL97* (Sheldrick, 2008), Fc^*^=kFc[1+0.001xFc^2^λ^3^/sin(2θ)]^-1/4^
Primary atom site location: structure-invariant direct methods	Extinction coefficient: 0.0066 (11)

### Special details

**Table:**

Geometry. All esds (except the esd in the dihedral angle between two l.s. planes) are estimated using the full covariance matrix. The cell esds are taken into account individually in the estimation of esds in distances, angles and torsion angles; correlations between esds in cell parameters are only used when they are defined by crystal symmetry. An approximate (isotropic) treatment of cell esds is used for estimating esds involving l.s. planes.
Refinement. Refinement of F^2^ against ALL reflections. The weighted R-factor wR and goodness of fit S are based on F^2^, conventional R-factors R are based on F, with F set to zero for negative F^2^. The threshold expression of F^2^ > 2sigma(F^2^) is used only for calculating R-factors(gt) etc. and is not relevant to the choice of reflections for refinement. R-factors based on F^2^ are statistically about twice as large as those based on F, and R- factors based on ALL data will be even larger.

### Fractional atomic coordinates and isotropic or equivalent isotropic displacement parameters (Å^2^)

**Table:**

	*x*	*y*	*z*	*U*_iso_*/*U*_eq_	
C1	0.36015 (14)	0.01198 (9)	0.26268 (15)	0.0391 (4)	
H7	0.4045	0.0419	0.3291	0.047*	
C2	0.23967 (14)	0.12916 (9)	0.20681 (14)	0.0373 (4)	
C3	0.39979 (15)	0.08608 (9)	0.07274 (15)	0.0425 (4)	
C4	0.23326 (14)	0.04423 (9)	0.24437 (14)	0.0372 (4)	
H8	0.1903	0.0165	0.1743	0.045*	
C5	0.16508 (15)	0.03406 (9)	0.36401 (14)	0.0419 (4)	
H6	0.1853	−0.0169	0.3975	0.050*	
C6	0.26422 (15)	0.15513 (10)	0.43883 (15)	0.0436 (4)	
C7	0.05756 (18)	0.12622 (12)	0.05276 (18)	0.0632 (6)	
H19	0.0897	0.0833	0.0166	0.076*	
C8	−0.0509 (2)	0.15353 (16)	0.0041 (2)	0.0809 (7)	
H20	−0.0919	0.1286	−0.0637	0.097*	
C9	−0.0985 (2)	0.21711 (15)	0.0550 (2)	0.0752 (7)	
H21	−0.1719	0.2354	0.0225	0.090*	
C10	−0.03787 (19)	0.25315 (12)	0.1531 (2)	0.0709 (6)	
H22	−0.0697	0.2968	0.1871	0.085*	
C11	0.07081 (17)	0.22610 (10)	0.20371 (19)	0.0544 (5)	
H23	0.1112	0.2516	0.2713	0.065*	
C12	0.11932 (15)	0.16153 (9)	0.15439 (14)	0.0401 (4)	
C13	0.36784 (14)	−0.07141 (9)	0.30018 (16)	0.0411 (4)	
C14	0.33752 (17)	−0.12905 (10)	0.2150 (2)	0.0562 (5)	
H9	0.3092	−0.1171	0.1324	0.067*	
C15	0.3491 (2)	−0.20481 (12)	0.2519 (3)	0.0792 (7)	
H12	0.3274	−0.2433	0.1940	0.095*	
C16	0.3920 (2)	−0.22366 (14)	0.3727 (3)	0.0882 (9)	
H13	0.4006	−0.2747	0.3964	0.106*	
C17	0.42204 (19)	−0.16715 (15)	0.4581 (3)	0.0788 (7)	
H11	0.4512	−0.1795	0.5403	0.095*	
C18	0.40914 (17)	−0.09161 (12)	0.42245 (19)	0.0594 (5)	
H10	0.4286	−0.0534	0.4817	0.071*	
C19	−0.0248 (2)	−0.02385 (13)	0.26918 (19)	0.0676 (6)	
H14	0.0207	−0.0640	0.2423	0.081*	
C20	0.03073 (16)	0.03569 (10)	0.33765 (15)	0.0461 (4)	
C21	−0.03979 (18)	0.09298 (11)	0.38007 (19)	0.0597 (5)	
H18	−0.0056	0.1327	0.4288	0.072*	
C22	−0.1615 (2)	0.09177 (16)	0.3504 (3)	0.0835 (8)	
H17	−0.2078	0.1310	0.3792	0.100*	
C23	−0.2142 (2)	0.0342 (2)	0.2799 (3)	0.0919 (9)	
H16	−0.2956	0.0347	0.2589	0.110*	
C24	−0.1464 (2)	−0.02467 (18)	0.2401 (2)	0.0863 (8)	
H15	−0.1819	−0.0650	0.1939	0.104*	
N1	0.41552 (14)	0.02324 (9)	0.14448 (14)	0.0510 (4)	
N2	0.32026 (12)	0.13779 (8)	0.10666 (13)	0.0413 (4)	
N3	0.28885 (13)	0.17073 (8)	0.31879 (12)	0.0438 (4)	
N4	0.20999 (14)	0.08804 (9)	0.45970 (14)	0.0523 (4)	
O1	0.45673 (11)	0.09641 (7)	−0.02172 (11)	0.0588 (4)	
O2	0.29450 (12)	0.19940 (7)	0.52711 (11)	0.0574 (4)	
O3	0.33428 (16)	0.17446 (9)	0.78386 (16)	0.0704 (4)	
H4	0.3149 (17)	0.2203 (12)	0.3069 (18)	0.065 (6)*	
H24	0.3154 (16)	0.1796 (11)	0.0634 (17)	0.053 (5)*	
H5	0.4604 (17)	−0.0120 (11)	0.1161 (18)	0.059 (6)*	
H3	0.1959 (18)	0.0816 (12)	0.538 (2)	0.068 (6)*	
H2	0.348 (2)	0.1705 (15)	0.705 (3)	0.105 (10)*	
H1	0.396 (3)	0.1510 (18)	0.834 (3)	0.134 (12)*	

### Atomic displacement parameters (Å^2^)

**Table:**

	*U*^11^	*U*^22^	*U*^33^	*U*^12^	*U*^13^	*U*^23^
C1	0.0430 (10)	0.0364 (9)	0.0385 (8)	0.0023 (7)	0.0078 (7)	0.0025 (7)
C2	0.0451 (10)	0.0350 (9)	0.0329 (8)	0.0041 (7)	0.0102 (7)	−0.0003 (7)
C3	0.0454 (10)	0.0415 (10)	0.0419 (9)	0.0079 (8)	0.0128 (8)	0.0058 (7)
C4	0.0441 (10)	0.0349 (9)	0.0337 (8)	0.0029 (7)	0.0087 (7)	−0.0011 (7)
C5	0.0520 (11)	0.0367 (9)	0.0385 (9)	0.0022 (8)	0.0129 (8)	0.0026 (7)
C6	0.0462 (10)	0.0479 (10)	0.0366 (9)	0.0083 (8)	0.0032 (7)	−0.0050 (8)
C7	0.0619 (13)	0.0773 (14)	0.0495 (11)	0.0218 (11)	−0.0001 (10)	−0.0104 (10)
C8	0.0638 (15)	0.112 (2)	0.0636 (13)	0.0229 (14)	−0.0117 (11)	−0.0120 (13)
C9	0.0529 (13)	0.0919 (17)	0.0806 (15)	0.0226 (12)	0.0045 (12)	0.0150 (14)
C10	0.0604 (14)	0.0555 (13)	0.0983 (17)	0.0202 (11)	0.0158 (12)	0.0005 (12)
C11	0.0513 (11)	0.0439 (10)	0.0688 (12)	0.0075 (9)	0.0096 (9)	−0.0036 (9)
C12	0.0429 (10)	0.0420 (9)	0.0368 (8)	0.0048 (7)	0.0118 (7)	0.0049 (7)
C13	0.0367 (9)	0.0379 (9)	0.0501 (10)	0.0040 (7)	0.0120 (7)	0.0069 (8)
C14	0.0589 (12)	0.0446 (11)	0.0673 (12)	−0.0003 (9)	0.0175 (10)	−0.0022 (9)
C15	0.0836 (17)	0.0423 (12)	0.117 (2)	−0.0061 (11)	0.0391 (15)	−0.0081 (13)
C16	0.0692 (16)	0.0487 (14)	0.153 (3)	0.0128 (12)	0.0479 (17)	0.0400 (17)
C17	0.0558 (14)	0.0805 (17)	0.1011 (18)	0.0085 (12)	0.0119 (12)	0.0540 (15)
C18	0.0563 (12)	0.0601 (12)	0.0611 (12)	−0.0037 (10)	0.0009 (10)	0.0207 (10)
C19	0.0715 (15)	0.0681 (14)	0.0666 (13)	−0.0176 (11)	0.0253 (11)	−0.0107 (11)
C20	0.0529 (11)	0.0454 (10)	0.0422 (9)	−0.0032 (8)	0.0172 (8)	0.0054 (8)
C21	0.0588 (13)	0.0535 (12)	0.0699 (12)	0.0029 (10)	0.0246 (10)	0.0060 (10)
C22	0.0590 (15)	0.0851 (18)	0.111 (2)	0.0151 (13)	0.0339 (14)	0.0259 (16)
C23	0.0548 (15)	0.132 (3)	0.0900 (18)	−0.0157 (17)	0.0133 (14)	0.0343 (18)
C24	0.0707 (17)	0.120 (2)	0.0700 (15)	−0.0420 (16)	0.0173 (13)	−0.0067 (14)
N1	0.0580 (10)	0.0447 (9)	0.0541 (9)	0.0191 (8)	0.0274 (8)	0.0148 (7)
N2	0.0495 (9)	0.0351 (8)	0.0415 (8)	0.0091 (7)	0.0166 (6)	0.0092 (6)
N3	0.0564 (9)	0.0365 (8)	0.0389 (8)	−0.0015 (7)	0.0056 (6)	−0.0019 (6)
N4	0.0663 (11)	0.0598 (10)	0.0321 (8)	−0.0081 (8)	0.0121 (7)	−0.0015 (7)
O1	0.0683 (9)	0.0564 (8)	0.0566 (8)	0.0213 (6)	0.0352 (7)	0.0193 (6)
O2	0.0704 (9)	0.0578 (8)	0.0431 (7)	0.0036 (7)	−0.0007 (6)	−0.0156 (6)
O3	0.0936 (12)	0.0656 (10)	0.0516 (9)	0.0181 (8)	0.0030 (8)	0.0027 (7)

### Geometric parameters (Å, °)

**Table:**

C1—N1	1.458 (2)	C11—H23	0.9300
C1—C13	1.512 (2)	C13—C14	1.377 (2)
C1—C4	1.540 (2)	C13—C18	1.384 (2)
C1—H7	0.9800	C14—C15	1.385 (3)
C2—N3	1.461 (2)	C14—H9	0.9300
C2—N2	1.462 (2)	C15—C16	1.370 (4)
C2—C12	1.536 (2)	C15—H12	0.9300
C2—C4	1.541 (2)	C16—C17	1.363 (4)
C3—O1	1.2460 (18)	C16—H13	0.9300
C3—N1	1.339 (2)	C17—C18	1.379 (3)
C3—N2	1.345 (2)	C17—H11	0.9300
C4—C5	1.546 (2)	C18—H10	0.9300
C4—H8	0.9800	C19—C24	1.385 (3)
C5—N4	1.446 (2)	C19—C20	1.389 (3)
C5—C20	1.523 (2)	C19—H14	0.9300
C5—H6	0.9800	C20—C21	1.379 (3)
C6—O2	1.2397 (19)	C21—C22	1.387 (3)
C6—N3	1.350 (2)	C21—H18	0.9300
C6—N4	1.351 (2)	C22—C23	1.362 (4)
C7—C8	1.376 (3)	C22—H17	0.9300
C7—C12	1.379 (2)	C23—C24	1.370 (4)
C7—H19	0.9300	C23—H16	0.9300
C8—C9	1.366 (3)	C24—H15	0.9300
C8—H20	0.9300	N1—H5	0.87 (2)
C9—C10	1.351 (3)	N2—H24	0.862 (19)
C9—H21	0.9300	N3—H4	0.93 (2)
C10—C11	1.384 (3)	N4—H3	0.86 (2)
C10—H22	0.9300	O3—H2	0.87 (3)
C11—C12	1.377 (2)	O3—H1	0.94 (3)
			
N1—C1—C13	109.63 (13)	C14—C13—C1	121.94 (16)
N1—C1—C4	107.75 (12)	C18—C13—C1	119.93 (16)
C13—C1—C4	114.79 (13)	C13—C14—C15	120.3 (2)
N1—C1—H7	108.2	C13—C14—H9	119.9
C13—C1—H7	108.2	C15—C14—H9	119.9
C4—C1—H7	108.2	C16—C15—C14	120.8 (2)
N3—C2—N2	108.53 (13)	C16—C15—H12	119.6
N3—C2—C12	112.11 (12)	C14—C15—H12	119.6
N2—C2—C12	106.73 (12)	C17—C16—C15	119.6 (2)
N3—C2—C4	107.08 (12)	C17—C16—H13	120.2
N2—C2—C4	109.39 (12)	C15—C16—H13	120.2
C12—C2—C4	112.92 (13)	C16—C17—C18	119.9 (2)
O1—C3—N1	121.35 (15)	C16—C17—H11	120.0
O1—C3—N2	121.18 (15)	C18—C17—H11	120.0
N1—C3—N2	117.47 (14)	C17—C18—C13	121.4 (2)
C1—C4—C2	108.80 (13)	C17—C18—H10	119.3
C1—C4—C5	112.20 (12)	C13—C18—H10	119.3
C2—C4—C5	111.06 (12)	C24—C19—C20	121.4 (2)
C1—C4—H8	108.2	C24—C19—H14	119.3
C2—C4—H8	108.2	C20—C19—H14	119.3
C5—C4—H8	108.2	C21—C20—C19	117.78 (19)
N4—C5—C20	113.82 (14)	C21—C20—C5	123.35 (17)
N4—C5—C4	109.13 (13)	C19—C20—C5	118.87 (17)
C20—C5—C4	113.95 (13)	C20—C21—C22	120.4 (2)
N4—C5—H6	106.5	C20—C21—H18	119.8
C20—C5—H6	106.5	C22—C21—H18	119.8
C4—C5—H6	106.5	C23—C22—C21	121.2 (2)
O2—C6—N3	121.10 (17)	C23—C22—H17	119.4
O2—C6—N4	121.38 (15)	C21—C22—H17	119.4
N3—C6—N4	117.46 (15)	C22—C23—C24	119.5 (2)
C8—C7—C12	121.00 (19)	C22—C23—H16	120.2
C8—C7—H19	119.5	C24—C23—H16	120.2
C12—C7—H19	119.5	C23—C24—C19	119.7 (2)
C9—C8—C7	120.2 (2)	C23—C24—H15	120.2
C9—C8—H20	119.9	C19—C24—H15	120.2
C7—C8—H20	119.9	C3—N1—C1	123.42 (14)
C10—C9—C8	119.4 (2)	C3—N1—H5	116.2 (13)
C10—C9—H21	120.3	C1—N1—H5	120.4 (13)
C8—C9—H21	120.3	C3—N2—C2	126.51 (14)
C9—C10—C11	121.1 (2)	C3—N2—H24	116.3 (12)
C9—C10—H22	119.5	C2—N2—H24	117.1 (12)
C11—C10—H22	119.5	C6—N3—C2	124.60 (15)
C12—C11—C10	120.20 (19)	C6—N3—H4	114.1 (12)
C12—C11—H23	119.9	C2—N3—H4	117.4 (12)
C10—C11—H23	119.9	C6—N4—C5	126.24 (14)
C11—C12—C7	118.07 (17)	C6—N4—H3	113.2 (14)
C11—C12—C2	122.34 (15)	C5—N4—H3	120.2 (14)
C7—C12—C2	119.58 (15)	H2—O3—H1	109 (3)
C14—C13—C18	118.12 (17)		
			
N1—C1—C4—C2	−57.64 (16)	C14—C15—C16—C17	1.0 (3)
C13—C1—C4—C2	179.93 (13)	C15—C16—C17—C18	−0.1 (3)
N1—C1—C4—C5	179.05 (13)	C16—C17—C18—C13	−1.1 (3)
C13—C1—C4—C5	56.63 (18)	C14—C13—C18—C17	1.3 (3)
N3—C2—C4—C1	−70.43 (15)	C1—C13—C18—C17	−177.18 (17)
N2—C2—C4—C1	47.01 (16)	C24—C19—C20—C21	2.3 (3)
C12—C2—C4—C1	165.70 (12)	C24—C19—C20—C5	−178.68 (18)
N3—C2—C4—C5	53.55 (17)	N4—C5—C20—C21	12.8 (2)
N2—C2—C4—C5	170.99 (13)	C4—C5—C20—C21	−113.21 (18)
C12—C2—C4—C5	−70.32 (16)	N4—C5—C20—C19	−166.15 (15)
C1—C4—C5—N4	73.88 (17)	C4—C5—C20—C19	67.8 (2)
C2—C4—C5—N4	−48.15 (18)	C19—C20—C21—C22	−2.4 (3)
C1—C4—C5—C20	−157.68 (14)	C5—C20—C21—C22	178.66 (17)
C2—C4—C5—C20	80.30 (17)	C20—C21—C22—C23	0.4 (3)
C12—C7—C8—C9	0.9 (4)	C21—C22—C23—C24	1.6 (4)
C7—C8—C9—C10	0.4 (4)	C22—C23—C24—C19	−1.7 (4)
C8—C9—C10—C11	−0.9 (4)	C20—C19—C24—C23	−0.3 (3)
C9—C10—C11—C12	0.1 (3)	O1—C3—N1—C1	173.66 (17)
C10—C11—C12—C7	1.2 (3)	N2—C3—N1—C1	−6.2 (3)
C10—C11—C12—C2	179.89 (17)	C13—C1—N1—C3	164.44 (16)
C8—C7—C12—C11	−1.7 (3)	C4—C1—N1—C3	38.9 (2)
C8—C7—C12—C2	179.58 (19)	O1—C3—N2—C2	173.39 (16)
N3—C2—C12—C11	4.8 (2)	N1—C3—N2—C2	−6.7 (3)
N2—C2—C12—C11	−113.91 (17)	N3—C2—N2—C3	100.86 (18)
C4—C2—C12—C11	125.86 (16)	C12—C2—N2—C3	−138.12 (16)
N3—C2—C12—C7	−176.50 (16)	C4—C2—N2—C3	−15.7 (2)
N2—C2—C12—C7	64.79 (19)	O2—C6—N3—C2	−169.06 (15)
C4—C2—C12—C7	−55.4 (2)	N4—C6—N3—C2	13.7 (2)
N1—C1—C13—C14	−47.6 (2)	N2—C2—N3—C6	−155.76 (15)
C4—C1—C13—C14	73.77 (19)	C12—C2—N3—C6	86.60 (19)
N1—C1—C13—C18	130.74 (17)	C4—C2—N3—C6	−37.8 (2)
C4—C1—C13—C18	−107.85 (18)	O2—C6—N4—C5	176.39 (16)
C18—C13—C14—C15	−0.3 (3)	N3—C6—N4—C5	−6.4 (3)
C1—C13—C14—C15	178.08 (17)	C20—C5—N4—C6	−103.71 (19)
C13—C14—C15—C16	−0.8 (3)	C4—C5—N4—C6	24.8 (2)

### Hydrogen-bond geometry (Å, °)

**Table:**

*D*—H···*A*	*D*—H	H···*A*	*D*···*A*	*D*—H···*A*
N1—H5···O1^i^	0.87 (2)	2.05 (2)	2.9098 (19)	169.5 (18)
N2—H24···O2^ii^	0.862 (19)	2.16 (2)	2.9774 (19)	157.9 (16)
N3—H4···O3^ii^	0.93 (2)	1.87 (2)	2.787 (2)	168.1 (18)
O3—H1···O1^iii^	0.94 (3)	1.88 (3)	2.747 (2)	152 (3)
O3—H2···O2	0.87 (3)	1.99 (3)	2.751 (2)	146 (2)

Symmetry codes: (i) −*x*+1, −*y*, −*z*; (ii) *x*, −*y*+1/2, *z*−1/2; (iii) *x*, *y*, *z*+1.
